# Improved tissue culture conditions for the emerging C_4_ model *Panicum hallii*

**DOI:** 10.1186/s12896-017-0359-0

**Published:** 2017-04-27

**Authors:** Joshua N. Grant, Jason N. Burris, C. Neal Stewart, Scott C. Lenaghan

**Affiliations:** 1 Department of Plant Science, University of Tennessee, 2431 Joe Johnson Drive, Knoxville, TN 37996 USA; 2 Department of Food Science, University of Tennessee, 2600 River Drive, Knoxville, TN 37996 USA; 3 Department of Mechanical, Aerospace, and Biomedical Engineering, University of Tennessee, 1512 Middle Drive, Knoxville, TN 37996 USA

**Keywords:** C_4_ model, Tissue culture, *Panicum hallii*, *Panicum virgatum*, Regeneration, Recalcitrance, Suspension culture

## Abstract

**Background:**

*Panicum hallii* Vasey (Hall’s panicgrass) is a compact, perennial C_4_ grass in the family Poaceae, which has potential to enable bioenergy research for switchgrass (*Panicum virgatum* L.). Unlike *P. hallii*, switchgrass has a large genome, allopolyploidy, self-incompatibility, a long life cycle, and large stature—all suboptimal traits for rapid genetics research. Herein we improved tissue culture methodologies for two inbred *P. hallii* populations: FIL2 and HAL2, to enable further development of *P. hallii* as a model C_4_ plant.

**Results:**

The optimal seed-derived callus induction medium was determined to be Murashige and Skoog (MS) medium supplemented with 40 mg L^−1^ L-cysteine, 300 mg L^−1^ L-proline, 3% sucrose, 1 g L^−1^ casein hydrolysate, 3 mg L^−1^ 2,4-dichlorophenoxyacetic acid (2,4-D), and 45 μg L^−1^ 6-benzylaminopurine (BAP), which resulted in callus induction of 51 ± 29% for FIL2 and 81 ± 19% for HAL2. The optimal inflorescence-derived callus induction was observed on MP medium (MS medium supplemented with 2 g L^−1^ L-proline, 3% maltose, 5 mg L^−1^ 2,4-D, and 500 μg L^−1^ BAP), resulting in callus induction of 100 ± 0.0% for FIL2 and 84 ± 2.4% for HAL2. Shoot regeneration rates of 11.5 ± 0.8 shoots/gram for FIL2 and 11.3 ± 0.6 shoots/gram for HAL2 were achieved using seed-induced callus, whereas shoot regeneration rates of 26.2 ± 2.6 shoots/gram for FIL2 and 29.3 ± 3.6 shoots/gram for HAL2 were achieved from inflorescence-induced callus. Further, cell suspension cultures of *P. hallii* were established from seed-derived callus, providing faster generation of callus tissue compared with culture using solidified media (1.41-fold increase for FIL2 and 3.00-fold increase for HAL2).

**Conclusions:**

Aside from abbreviated tissue culture times from callus induction to plant regeneration for HAL2, we noted no apparent differences between FIL2 and HAL2 populations in tissue culture performance. For both populations, the cell suspension cultures outperformed tissue cultures on solidified media. Using the methods developed in this work, *P. hallii* callus was induced from seeds immediately after harvest in a shorter time and with higher frequencies than switchgrass. For clonal propagation, *P. hallii* callus was established from R1 inflorescences, similar to switchgrass, which further strengthens the potential of this plant as a C_4_ model for genetic studies. The rapid cycling (seed-to-seed time) and ease of culture, further demonstrate the potential utility of *P. hallii* as a C_4_ model plant.

**Electronic supplementary material:**

The online version of this article (doi:10.1186/s12896-017-0359-0) contains supplementary material, which is available to authorized users.

## Background

Switchgrass, *Panicum virgatum* L., is a perennial C_4_ grass native to North America, which has shown promise as a cellulosic bioenergy feedstock [1]. As a feedstock, switchgrass is attractive in that it produces high biomass [2] with relatively low farmer input in a wide range of temperate climates [3]. The bioenergy potential of switchgrass has led to the development of numerous tissue culture and transformation protocols [4–11], along with a draft genome available from the United States Department of Energy (DOE) Joint Genome Institute (JGI, http://jgi.doe.gov/data-and-tools/genome-portal/). Transgenic switchgrass plants have been developed for improved cell wall biosynthesis traits for biofuel production, for example, the overexpression of transcription factors [12] and the use of RNAi-mediated knockdowns [13]. However, like many crops, switchgrass transformation, while reliable, takes around six months from callus induction to regeneration of plants [9]. Further, switchgrass is self-incompatible, which, along with its large genome [14] and allopolyploidy result in complicated genetic analysis scenarios [15]. Therefore, a reverse genetics pipeline could be enhanced by the identification of an appropriate fast cycling C_4_ model plant to speed the development of the next-generation switchgrass.

As a potential C_4_ model plant, *P. hallii* displays many desirable qualities: it is small in stature (average mature heights of accessions are 35.6–65.7 cm), has a small genome (453–550 Mb), and a rapid life cycle (seed-to-seed time of 40–90 d) [16, 17]. Further, *P. hallii* can produce somatic embryogenic callus from seed within 35–50 d, compared to 120 d for switchgrass [18]. Previous studies on *P. hallii* have focused on the development of microsatellite markers [19], analysis of gene expression and transcriptomics [20], exploration of biodiversity within the species [17], and the genetic divergence of ecotypes [16]. Additionally, a tissue culture [21] and regeneration system [22] for mature seeds (>1 year old) of *P. hallii* has been developed and compared with other *Panicum* species. The goal of the current study was to develop facile and robust tissue culture methodologies for *P. hallii* using inflorescences, fresh seeds (<6 months old), and cell suspension cultures.

## Methods

### Plant material and reagents

Seeds from inbred populations of *P. hallii* var. *filipes* (Scribn.) Waller (PAHAF) and *P. hallii* Vasey var. *hallii* (PAHAH), designated FIL2 and HAL2 were kindly donated by Dr. Tom Juenger and colleagues at the University of Texas at Austin [16]. Plants generated from these seeds were grown in greenhouses, selfed, and their progeny yielded seeds for subsequent experiments. All plants were grown under a 16 h photoperiod, and mature panicles were lightly shaken to assist self-fertilization and seed set. Seeds were collected and plated on various media in a randomized block design. For inflorescence-derived callus, inflorescences were collected from plants at the onset of bolting before panicle emergence. Callus generated from inflorescences of a tissue culture elite switchgrass control, Performer 605 (PVP-605), was used for comparison in all experiments.

Basal media components complete with vitamins of Murashige and Skoog (MS), Kao & Michayluk (KM8), and Chu’s N6 (NB) were obtained from PhytoTechnology Laboratories (Shawnee Mission, KS, USA). Media components for LP9 [7] and AA [23] were obtained from Sigma-Aldrich (St. Louis, MO, USA). All media components were mixed and contained 30 g L^−1^ of sucrose (Thermo Fisher Scientific, Waltham, MA, USA) or maltose (Sigma-Aldrich, St. Louis, MO, USA). The plant hormones used in the following experiments were 2,4-dichlorophenoxyacetic acid (2,4-D) (PhytoTechnology Laboratories), 6-benzylaminopurine (BAP) (PhytoTechnology Laboratories, Shawnee Mission, KS, USA), and gibberellic acid (GA3) (Sigma-Aldrich, St. Louis, MO, USA). For solidified media, Phytagel (3 g L^−1^, Sigma-Aldrich, St. Louis, MO, USA) was added before autoclaving, and 15 mL were poured into Petri dishes and solidified under aseptic conditions in a laminar flow hood.

### Seed germination and sterilization

Seeds immediately harvested from greenhouse grown plants and seeds stored for > 1 year were tested for germination efficiency with and without seed coat removal [21]. To remove the seed coat, chaff was manually separated from seeds, and 300 grit sandpaper was used to abrade the seed coat (Juenger, personal communication). Three replicates consisting of 33 seeds per plate were used to determine the germination efficiency. Prior to plating on MS medium with no sugar or hormones (Diet-MS), seeds were suspended in 0.5 mL of either sterile water or a filter-sterilized solution of 1.44 μM GA3. Seeds were then pipetted onto plates and incubated at 24 °C in either the dark or the light. Coleoptile emergence was monitored weekly for three weeks; germination frequency was calculated as the number of seeds with an emerging coleoptile divided by the total number of seeds on the plate. After determining the best method for germination, surface sterilization methods were tested using two treatments: a combination of 5% dilution of commercial sodium hypochlorite bleach and 70% ethanol (Treatment 1, Juenger, personal communication) or a modified chlorine gas protocol (Treatment 2, [23]). For Treatment 1, seeds were immersed in 5% bleach and agitated for one minute, then transferred to 70% ethanol and agitated for one minute before being washed five times with sterile water. For Treatment 2, seeds were placed into 1.5 mL microfuge tubes up to the 0.1 mL mark. Tubes, with their caps open, were then placed in an air-tight chamber with 33 mL of bleach in a fume hood. Next, 1 mL of 12 N HCl was added to the bleach before sealing the air-tight chamber. Seeds were left in the chamber for 16 h before being transferred to a laminar flow hood for de-fumigation for 15 min. Seeds were then immediately placed onto Diet-MSO. Seed sterilization efficiency was determined by calculating both germination frequency and scoring the seeds for the presence or absence of contamination around an individual seed after six weeks. To determine significance between the two treatments, Student’s T-tests were conducted, as described in the statistical analysis.

### Media optimization

We assessed the performance of *P. hallii* callus induction and proliferation using media defined from the monocot tissue culture literature: AA [24] and KM8 [25], LP9 [8], MS [26], MS-OG [27], MS-BH [28], MS-PM [29], MS-SC [30], MS-SEO [21], MP [6], and MP-PAH, a novel medium developed in this work based on preliminary experimentation with *P. hallii* [see Additional file 1]. Germination efficiency, percent callus induction, callus type (I-IV), callus proliferation, and regeneration frequency were determined for each medium. Callus induction frequency was calculated in triplicate using 33 seeds per plate per medium. Plates were examined weekly for callus formation from each individual seed, and the number of seeds producing callus was recorded. The type of callus was scored on the following scale: type I was hard, compact, and white; type II was friable, hard, and light yellow; type III was fast-growing, mucilaginous, and yellow to white; type IV was spongey and slow-growing. Callus proliferation at a range of temperatures (20, 24, 28, 32, and 36 °C) was measured on four replicates, each containing 3 g of callus. The fresh weight of callus was taken 4 weeks after induction. Callus induced on each medium was subdivided into three replicates of 3 g each to conduct growth rate analysis. Callus growth was measured after four weeks by mass gained. The plant regeneration experiment tallied the number of shoots from three replicate plates, each containing 1 g callus, by medium [see Additional file 2]. All regeneration media were based on MS at pH 5.8, with a few modifications: REG contained 30 g L^−1^ maltose, 40 mg L^−1^ BAP, 485 μg L^−1^ GA3; REG-SEO contained 30 g L^−1^ maltose, 4.8 mg L^−1^ naphathalene acetic acid (NAA), and 990 μg L^−1^ GA3 REG-R contained 4.8 mg L^−1^ NAA and 485 μg L^−1^ GA3; REG-SEO-R contained 4.8 mg L^−1^ NAA; diet-MS contained no sugars or hormones. Regeneration frequency was calculated as number of shoots per callus piece and number of shoots per gram. The optimal medium was determined by evaluating the performance of each medium for callus induction rate, callus type, and plant regeneration.

### Suspension culture

All media used in the tissue culture experiments were evaluated for establishment of suspension cultures. Suspension cultures were initiated by placing 2.5 g of macerated, heterogeneous callus into 100 mL flasks, containing 30 mL of each medium type, with weekly subcultures for 4 weeks until suspension cultures were established. Initial subcultures were conducted by allowing cells to settle at room temperature for about 10 min, removing supernatant, and resuspending them in 30 mL of fresh medium. Flasks containing 30 mL of medium with no tissue were used as a control for media evaporation, with media being exchanged weekly. All experiments were performed in triplicate. Cell suspension characteristics were analyzed using the following methods: dissimilation curves to measure growth characteristics [31], packed cell volume to quantify total growth after 30 d [32], cell viability through fluorescein diacetate-propidium iodide (FDA-PI) simultaneous double-staining [33], and cell size distribution using image analysis of micrographs [34].

Dissimilation curves were measured for 30 d by comparing the daily evaporation relative to the sentinel flasks to the daily mass change in the inoculated flasks. The average evaporation of each control flask was taken daily and added back to the difference between the previous day and current day mass for each corresponding media. *D* = (*S*_*P*_ − *S*_*C*_) + (*C*_*P*_ − *C*_*C*_), where D is the dissimilation of carbon from the sugar source, S_C_ is the sample’s current day mass, S_P_ is the sample’s previous day mass, C_P_ is the control’s previous day mass, C_C_ is the control’s current day mass. Subcultures were made every two weeks by transferring to a 50 mL Falcon tube, centrifuging for 10 min at 150 × *g* at room temperature, removing spent medium, and resuspending with 30 mL of medium. Packed cell volume (PCV) was measured by taking three 1 mL aliquots for each medium after 30 d and centrifugation for 10 min at 150 × *g* at room temperature and measuring the volume of the cell pellet. Cell viability was confirmed after 30 d by taking 1 mL aliquots from each flask and staining with 10 μL of a 0.1% FDA solution and 5 μL of a 0.2% PI solution. Eppendorf tubes were covered with aluminum foil and vortexed on low speed for 30 sec. After incubation in the dark for 5 min two 10 μL replicates were examined on a hemocytometer. This method generated three biological replicates with two technical replicates for each treatment. Cell viability was calculated as a percentage of live cells out of the total number of cells. Cell size distribution was calculated by placing 10 mL of cell suspensions from each flask into a canted 25 cm^2^ flat-bottomed flask and observing 100 cells using an inverted microscope. Then, 100 cells were measured using the image analysis software package FIJI [35]. Cells were analyzed for total area and length to width ratio. Length to width ratio was calculated by taking the larger measurement as length and the smaller measurement as width.

Regeneration of plants from suspension cells was conducted by two treatment methods. Treatment 1 involved removing all media from suspension cells, washing the cells three times in a medium containing no hormones followed by resuspension of cells in one of two regeneration media: REG or REG-SEO. A secondary regeneration experiment was performed by transferring intact callus pieces back onto solid MS-OG for two weeks before attempting regeneration on solidified medium. Populations were analyzed separately and treatments were compared using a two-way ANOVA controlling for initiation medium and regeneration medium.

### Direct comparison with published methods [21, 22]

The tissue culture methods developed in this work were directly compared to previously published methods across three parameters: callus induction, callus proliferation, and regeneration. Seeds stored for greater than one year and seeds immediately harvested from the greenhouse were sterilized using chlorine gas and plated onto either MS-OG or MS-SEO and analyzed weekly for eight weeks to score callus induction. Callus was then weighed and checked for proliferation by weighing callus at each subculture for four weeks. Eleven pieces of type II callus were selected for regeneration from each medium. Regeneration was scored weekly for four weeks. All statistics were analyzed by population type and controlling for medium using a one-way ANOVA. If significant differences were observed in the ANOVA at *p* = 0.05, then mean separation was calculated using Tukey’s Honest Significant Difference.

### Callus induction from inflorescences

Callus was induced from inflorescences using previously described methods [36], in which immature inflorescences were surface sterilized using 5% dilution of commercial bleach for 30 min and 70% ethanol for 10 min, before being cultured for two weeks on MSB (MS supplemented with 4 mg L^−1^ BAP, 3% maltose, and 2 g L^−1^ Phytagel). After two weeks, pre-cultured inflorescences were chopped into < 5 mm pieces and transferred onto MP medium [6] or MS-OG medium, and callus was transferred bi-weekly after an initial four weeks of culture. After a total of eight weeks, eleven callus pieces were weighed and placed onto REG medium to determine the regeneration efficiency.

### Statistical analysis

All statistical analysis was performed using R 3.3.0 (R Core Team, Vienna, Austria). Tukey’s Honest Significant Difference was calculated using the package AGRICOLAE.

## Results and discussion

### Seed germination and sterilization

Grass species typically have mechanisms of seed dormancy [37], which often require seed coat scarification to break dormancy [38]. The most effective method for breaking seed dormancy of HAL2 seeds was found to be the removal of the seed coat with 300-grit sandpaper and germination in the dark (45.8 ± 2.4%; *p <* 0.05). Seed coat removal did not affect germination of FIL2 seeds. The chlorine gas sterilization procedure appeared to be effective each instance, whereas minor microbial contamination was observed in cultures after the bleach treatment. Therefore, chlorine gas was used subsequently for seed sterilization. Our standard germination procedure was established to remove seed coats from HAL2, but not from FIL2 before sterilizing with chlorine gas, followed by germination in the dark.

### Media optimization

**Fig. 1:**
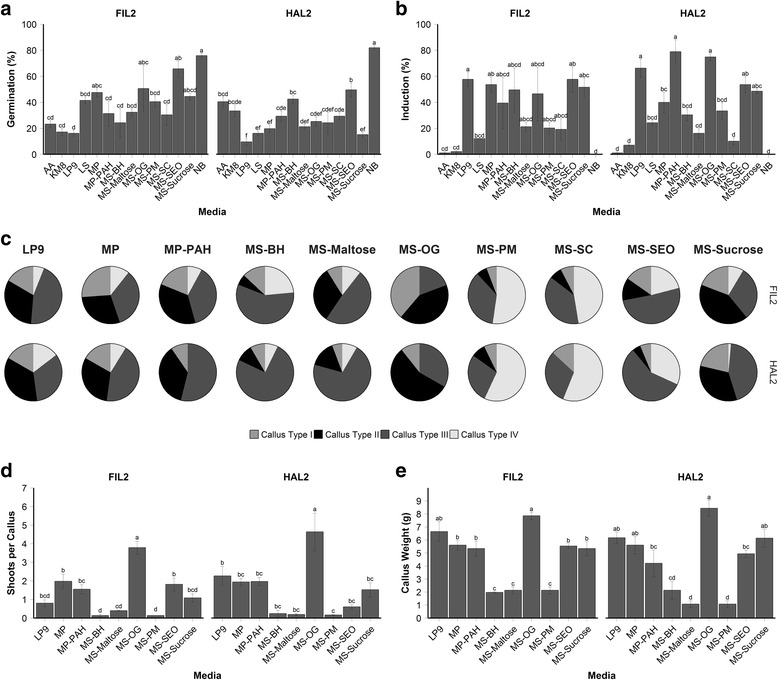
Results from media screen for tissue culture of *P. hallii*. **a** Effect of media on germination. Data represent three replicates of 33 seeds per plate. **b** Effect of media type on callus induction. **c** Types of callus from each medium. **d** Regeneration effect scored as shoots per callus piece for callus induced on each medium. Data represent three replicates of one gram each of callus (7–11 pieces). **e** Callus proliferation measured in grams for each medium. Data represent three replicates of two grams of callus. **a**, **b**, **d**, **e**) Populations were analyzed separately under a one-way ANOVA controlling for medium. ANOVA tests showed differences among treatments for both HAL2 and FIL2 (*p* *<* 0.01). Mean separation was analyzed with Tukey’s HSD, error bars represent the standard error of the mean

Experiments with various media indicated that NB medium promoted germination in HAL2 better than any other media type (Fig. 1a), with a rate 81.8 ± 1.7%, *p <* 0.05. However, seeds plated on NB failed to produce any callus (Fig. 1b). Seeds germinated on LP9 at 19.7 ± 3.9% (FIL2) and 10.1 ± 2.3% (HAL2), whereas callus was induced at 54.6 ± 12.0% (FIL2) and 64.1 ± 3.6% (HAL2); seeds germinated on MS-OG at 67.7 ± 2.7% (FIL2) and 17.2 ± 7.9% (HAL2) and callus was induced at 67.7 ± 2.7% (FIL2) and 81.8 ± 8.0% (HAL2). Seeds placed on MS-SEO had a high germination rate (50.5 ± 10.7% for FIL2; 45.5 ± 3.5% for HAL2), and a high induction rate (52.5 ± 5.3% for FIL2; 53.5 ± 6.1% for HAL2).

Next, callus type was scored and calculated as a percent of total callus induced for each media type (Fig. 1c). The apparent best medium for type II callus induction for FIL2 was MS-Sucrose (66.7 ± 1.7%) with a *p <* 0.05. The top performers of type II callus induction for HAL2 was MS-OG (70.7 ± 23.5%), MS-Sucrose (67.7 ± 3.6%), MP (53.0 ± 7.6%), and LP9 (42.3 ± 2.6%), with no significant differences among those treatments. No type IV callus was induced during this experiment, so the analysis only focused on callus types I, II, and III. For the next experiments, only LP9, MP, MP-PAH, MS-BH, MS-Maltose, MS-OG, MS-PM, MS-SEO, and MS-Sucrose were selected, since they resulted in the production of type II callus in both populations. In addition, the optimal temperature of callus production was 24–28 °C using MS-OG medium with a significant increase (*p <* 0.05) in mass of 2.14 ± 0.17 g (FIL2) and 2.36 ± 0.22 g (HAL2) compared to other temperatures tested.

More shoots per callus were produced in MS-OG medium: 3.8 ± 0.3 shoots per callus for FIL2 and 4.6 ± 1.0 shoots per callus for HAL2(Fig. 1d), which was significantly different from all other treatments (*p <* 0.05). MS-OG and LP9 media were optimal for FIL2 callus growth (7.9 ± 0.3 g and 6.2 ± 0.44 g, respectively). HAL2 callus responded to multiple media with no significant difference among the top four media: MS-OG, LP9, MP, and MS-Sucrose (Fig. 1e). Even though MS-OG medium was equivalent to those media just listed, it was superior in type II callus induction, and resulted ultimately in more regenerated shoots than the other media tested. Therefore, we chose MS-OG medium for subsequent experiments.

MS-OG medium was used then to test effects of various 2,4 D concentrations on callus growth [see Additional file 3: Figure S1. After 35 d, 0.75 mg L^−1^ 2,4-D, with callus subcultured weekly, performed better than all other auxin treatments for FIL2, which produced a callus area of 9.6 ± 1.2 cm^2^ when comparing populations separately under a one-way ANOVA controlling for treatment and analyzed at *p <* 0.05. However, HAL2 produced the same callus areas under treatments of 0.75 mg L^−1^ 2,4-D auxin with weekly callus subculture (8.7 ± 1.3 cm^2^), 3 mg L^−1^ 2,4-D auxin with bi-weekly callus subcultures (8.8 ± 1.4 cm^2^), and 3 mg L^−1^ 2,4-D with no callus subcultures (8.8 ± 1.3 cm^2^). HAL2 callus generated from the 0.37 mg L^−1^ 2,4-D auxin treatment was derived mainly from the coleoptile, therefore these results might have skewed the analysis. The treatment of 0.75 mg L^−1^ 2,4-D subcultured weekly led to increased callus production in FIL2 (9.6 ± 1.2 g) [see Additional file 3a], but there was no significant difference for this treatment and the 3 mg L^−1^ 2,4-D treatment for HAL2 (6.8 ± 1.4 g, FIL2; 8.7 ± 0.7 g, HAL2). Analysis of the callus type induced for each treatment [see Additional file 3b] indicated that after 35 d, the highest percentage of type II callus was obtained using the 3 mg L^−1^ 2,4-D auxin concentration (23.7 ± 2.3%, FIL2; 24.7 ± 2.4%, HAL2) regardless of subculture frequency. Most callus induced by the 0.75 mg L^−1^ 2,4-D treatment, subcultured weekly, was type III callus (88.1 ± 7.5%, FIL2; 73.3 ± 6.6%, HAL2). Therefore, the optimal protocol for tissue culture of *P. hallii* was to induce callus for two weeks on MS-OG containing 3 mg L^−1^ 2,4-D auxin, and then subculture bi-weekly indefinitely on the same medium.

The type of callus (I-IV) is perhaps the most important factor in tissue culture methods. In grasses, type II callus has optimal embryogenic capacity [7, 39]. In our experiments, we determined that two callus types readily produced shoots: type I and type II. Type III callus rarely led to plant regeneration and type IV callus never regenerated [see Additional file 4a]. The auxin 2,4-D is used in the tissue culture of grass species in varying concentrations: 20 mg L^−1^ for *Paspalum scrobiculatum* [40], 5 mg L^−1^ for switchgrass [7], and 10 mg L^−1^ for *Panicum maximum* [41], thus our results are on the low end of the requirement for panicoid grasses.

Prolonged subculturing of HAL2 callus introduced a mucilaginous covering of callus cultures after twenty weeks that appeared to be associated with decreased callus proliferation [see Additional file 5]. While HAL2 callus proliferated more quickly than FIL2, it also declined in proliferation between the 18th and 20th weeks [see Additional file 5], suggesting that the tissue should not be used after this time. FIL2 callus biomass doubling per week after week 24, while HAL2 began doubling in biomass after week 14.

### Suspension culture

**Fig. 2:**
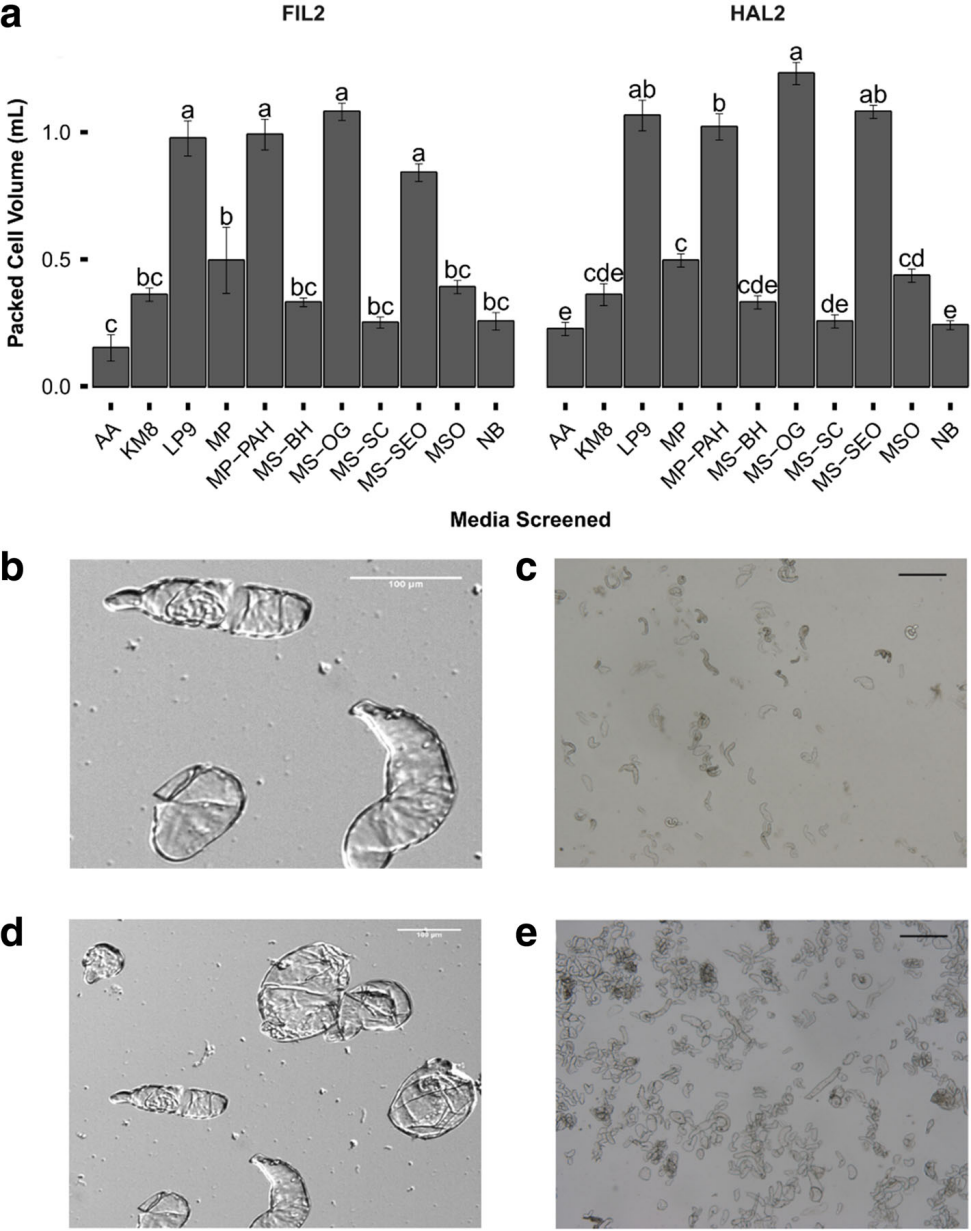
Analysis of packed cell volume (PCV) and cell morphology of cell suspension cultures of *P. hallii*. **a** Packed cell volume in mL of three replicates of 1.5 mL suspensions. Populations were analyzed separately under a one-way ANOVA controlling for medium. ANOVA test showed differences among treatments (*p <* 0.01). Mean separation was analyzed using Tukey’s HSD. Error bars represent the standard error of the mean. **b & c** FIL2 suspension cells. **d** & **e** HAL2 suspension cells. **b**-**e** Scale bars represent 100 μm

Dissimilation curve data [see Additional file 6] generated from suspension cultures established on each medium from the earlier screen demonstrated that MS-OG provided the best tissue growth. PCV data indicated that MS-OG was the optimal medium for suspension cultures (Fig. 2a). MS-OG appeared to be ineffective just after culture establishment, however, MS-OG enabled cultures to metabolize the most amount of carbon when compared with cultures on other media after 30 d of culture (3.84 ± 0.2 g, FIL2; 4.58 ± 0.3 g, HAL2). Further analysis of the packed cell volume (Fig. 2a) indicated that MS-OG (0.72 ± .023 mL), LP9 (0.66 ± 0.041 mL), MP-PAH (0.65 ± .046 mL), & MS-SEO (0.56 ± 0.023 mL) were not significantly different for the FIL2 population while suspensions maintained in MS-OG had the greatest packed cell volume for the HAL2 population at 0.82 ± 0.029 mL (*p <* 0.05). Dual staining with PI-FDA [see Additional file 7] indicated that MS-OG had the highest viability at the tested time-point for FIL2 (57.2 ± 3.4%, *p <* 0.05) and that there was no significant difference in LP9 (42.1 ± 3.6%), MP (44.7 ± 2.6%), MP-PAH (44.3 ± 3.1%), and MS-OG (48.0 ± 2.1%) for the HAL2 population. Unfortunately, plant regeneration from suspension culture in liquid medium was not observed in any treatments. However, shoot regeneration was observed when callus was re-established post cell culture by placing suspension cultures onto their corresponding medium followed by transfer to regeneration medium.

Suspension cultures allow for the generation of clonal variation within a single genotype [42] more quickly than tissue culture. Plant suspension cultures provide both faster growth than tissue culture and the ability for simple production, isolation, and purification of foreign proteins [43]. Suspension cultures can be synchronized [44] to obtain a homologous population of cells, thereby allowing experimentation on cell physiology, biochemistry, and metabolic events at the cellular level. A cell suspension culture can also aid in mutagenesis studies using CRISPR/Cas9 [45] or chemicals such as ethyl methanesulfonate [46]. The only downside to the system proposed here is that plant regeneration cannot occur directly from suspension cultures, which requires an extra solidified tissue culture step prior to plant regeneration.

### Direct comparison with published methods [21, 22]

**Fig. 3:**
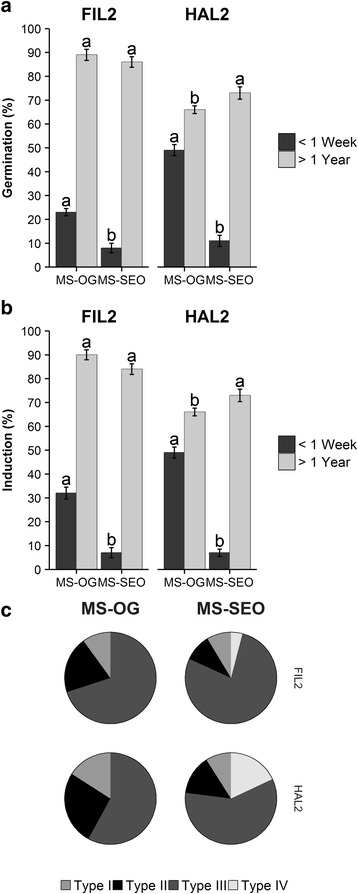
Comparison of tissue culture of *P. hallii* by MS-SEO and MS-OG media. **a** Germination results. **b** Callus induction results. **c** Pie graph showing type of callus induced. Populations were analyzed separately under a one-way ANOVA controlling for medium (*p <* 0.05). Mean separation was analyzed using Tukey’s HSD. Data represent ten replicates of ten seeds per replicate. Error bars represent the standard error of the mean

Seeds stored over a year were not significantly different in either germination frequency or callus induction rates between MS-OG and MS-SEO for FIL2 when compared via a Student’s *t*-test at *p <* 0.05, however HAL2 seeds aged > 1 year germinated and induced callus more frequently on MS-SEO. When populations were analyzed separately under a two-way ANOVA controlling for seed age and medium, germination and induction rates for seeds aged > 1 year were statistically similar for FIL2 yet statistically different for HAL2 when compared at *p <* 0.05 (Fig. 3a&b). Seeds immediately harvested from the greenhouse were statistically different regardless of population for either medium, with MS-OG consistently outperforming MS-SEO in both germination and callus induction: FIL2 germination rates increased from 8.0 ± 2.0% for MS-SEO to 23 ± 1.5% for MS-OG and HAL2 germination rates increased from 11 ± 2.3% for MS-SEO to 49 ± 2.3% for MS-OG, while FIL2 induction rates increased from 7.0 ± 2.1% for MS-SEO to 32 ± 2.5% for MS-OG and HAL2 induction rates increased from 10 ± 1.5% for MS-SEO 49 ± 2.3% for MS-OG (Fig. 3a&b). L-proline has been shown to promote somatic embryogenesis in maize [47, 48] and rice [49], and MS-OG contains 300 mg L^−1^ L-proline while MS-SEO contains none. There was no statistical difference among the callus types generated in MS-OG at either seed age (Fig. 3c). While the freshly harvested seed callus induction rates (51 ± 29% for FIL2 and 81 ± 19% for HAL2) were not as high as those previously published on mature seeds (49.9% for accession CPI.68864 and 96.7% for accession 85 B-1) [21], the method developed in this work allowed for seeds to be used within a week of harvest. The seeds used in previous studies were obtained from the National Institute of Livestock and Grassland Science, Tochigi, Japan and had been preserved at 4 °C for an undisclosed amount of time [21]. Seed age has been documented as affecting germination [50]. Since *P. hallii* was evaluated for use as a model system, a yearlong delay to gain an incrementally higher germination rates is not feasible.

A shoot regeneration screen [see Additional file 4a] indicated that REG and Diet-MS were the best media for shoot and root regeneration, respectively, regardless of either callus type I or II. Student’s *t-*test indicated that there was no significant difference between FIL2 and HAL2 when evaluated at *p <* 0.05. However, callus type did differ significantly within populations when evaluated with Student’s *t-*test at *p <* 0.05. Callus type I was able to induce shoots on REG at 27 ± 1.7 shoots/callus for FIL2 and 26 ± 1.73 shoots/callus for HAL2, and callus type II was able to induce shoots on REG at 3.3 ± 0.5 shoots/callus for FIL2 and 4.2 ± 0.5 shoots/callus for HAL2. Shoot regeneration on REG outperformed the other medium in this experiment, as REG-SEO was only able to produce 0.92 ± 0.2 shoots/callus FIL2 and 0.92 ± 0.1 shoots/callus for HAL2 for callus type II, which was significantly less (*p <* 0.05) than shoot regeneration on REG when populations and callus types were analyzed separately under a one-way ANOVA controlling for regeneration medium ([see Additional file 4a]. For root regeneration [see Additional file 4b], Diet-MSO optimally induced roots compared with other media tested, with a rooting frequency of 100 ± 0% for all callus with shoots for both populations. The data indicated that REG medium was statistically better at shoot induction than REG-SEO, and that Diet-MSO was statistically better at rooting than compared to either other medium when compared at *p <* 0.05.

For MS-SEO, some germinating seeds did not produce callus; conversely for MS-OG, callus was induced from seeds with no germination. Seed-derived callus for most grass species tend to produce callus from a germinated seed, such as with *Poa pratensis* [51]. The ability for the callus to be induced without seed germination may occur from endosperm tissue as seen in rice [52] and ryegrass [25]. However, callus from this source can be maintained and plants regenerated similarly to meristem-derived callus. The lack of any endosperm-derived callus in MS-SEO may indicate that one of the medium components of MS-OG is necessary to initiate endosperm-derived callus, but this could also be the result of the high ratio of auxin to cytokinin found in MS-OG.

### Callus induction from inflorescences

**Fig. 4:**
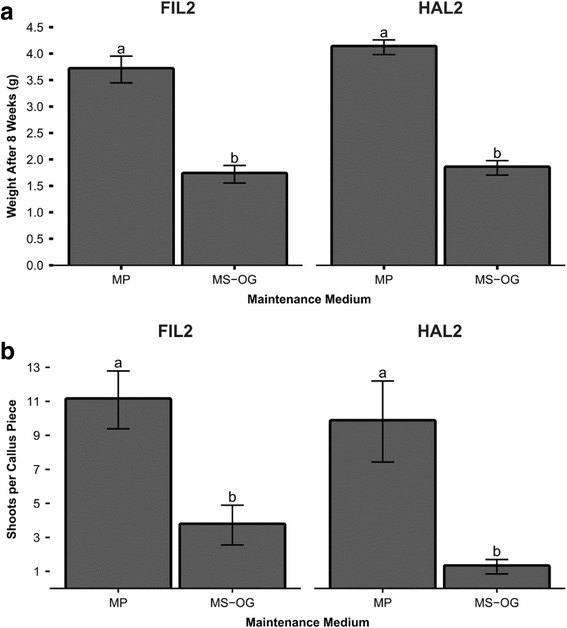
Analysis of tissue culture of *P. hallii* from immature inflorescences. **a** Callus weight at 8 weeks on maintenance medium. Data represent ten replicates of ten callus pieces per replicate. **b** Shoots per callus piece on REG medium. Data represent ten replicates of ten callus pieces per replicate. Populations were analyzed separately under a one-way ANOVA controlling for medium (*p <* 0.05). Mean separation was analyzed using Tukey’s HSD. Error bars represent the standard error of the mean

Callus induced from inflorescences performed significantly better (*p <* 0.05) when placed onto MP media under a one-way ANOVA controlling for medium (Fig. 4a), with FIL2 proliferating 3.7 ± 0.3 g additional weight for MP and only 1.7 ± 0.2 g for MS-OG and HAL2 producing 4.1 ± 0.1 g for MP and only 1.8 ± 0.1 g for MS-OG. MP was further confirmed as a better medium in allowing more shoots per callus piece to be induced (Fig. 4b): FIL2 yielded 11 ± 1.7 shoots per callus piece for MP and 3.7 ± 2.3 shoots per callus piece for MS-OG while HAL2 trended similarly with 9.8 ± 2.4 shoots per callus piece for MP and 1.3 ± 0.4 shoots per callus piece for MS-OG (*p <* 0.05). High levels of L-proline are commonly used in media maintaining inflorescence callus [47, 53]. MP contains 2 g L^−1^ of L-proline, while MS-OG contains 300 mg L^−1^. In addition, further experimentation should utilize callus induced from inflorescences, as this callus would be genotypically identical to the mother plant as opposed to seeds, which will have genetic variability.

## Conclusions

Both inbred populations of *P. hallii* can be cultured using semi-solid medium or liquid suspension cultures. The best medium for tissue or suspension culture for both populations was MS-OG. These cultures can undergo shoot regeneration on semi-solidified REG medium as quickly as one week for HAL2 and two weeks for FIL2. Root induction occurs with ease when Diet-MSO is used as rooting medium, with 100% of plantlets producing roots. Therefore, the speed with which our system can produce callus from both freshly harvested seed and inflorescences further demonstrates the potential of *P. hallii* as a model C_4_ plant. Additionally, this tissue culture procedure can be used to develop a transformation system in which seeds immediately harvested from the greenhouse or inflorescences cut from the plants can be used as explants, thus greatly increasing the speed of experiments. The specific impact of this work is the increased speed with which callus can be generated from either speed or plants.

## Additional files

Additional file 1: Table S1. Definition of all media used for callus induction. (PDF 121 kb)

Additional file 2: Table S2. A comparison of regeneration media used. (PDF 10 kb)

Additional file 3: Figure S1. Effect of 2,4-D on callus growth and type. (a) Total weight in grams of callus after 35 days of 2,4-D treatment. (b) Type of callus induced on each treatment. Data represent three replicates of 33 callus pieces per replicate. ANOVA test showed differences (*p <* 0.05). Mean separation was analyzed using Tukey’s HSD. Error bars represent the standard error of the mean. (PDF 300 kb)

Additional file 4: Figure S2. Comparison of callus regeneration by callus type and regeneration media. (a) Regeneration efficiency by callus type. (b) Rooting efficiency based on callus types and regeneration media. Populations and callus types were analyzed separately under a one-way ANOVA controlling for shoot regeneration medium. Mean separation was analyzed using Tukey’s HSD. Error bars represent the standard error of the mean. (PDF 340 kb)

Additional file 5: Figure S3. A comparison of FIL2 and HAL2 callus weight change after prolonged culture on MS-OG media. Each week was analyzed separately under a one-way ANOVA controlling for population. ANOVA test showing differences among populations are marked with an asterisk (*p <* 0.01). These data represent ten replicates of three grams of callus at each subculture. (PDF 157 kb)

Additional file 6: Figure S4. Dissimilation curve of suspension cell cultures of P. hallii. Each point represents one replicate of each measurement. (PDF 131 kb)

Additional file 7: Figure S5. Cell viability of cell suspension cultures as measured by dual staining with FDA and PI. Populations were analyzed separately under a one-way ANOVA controlling for medium (*p <* 0.05). Mean separation was analyzed using Tukey’s HSD. Data represent two technical replicates of three flasks. Error bars represent the standard error of the mean. (PDF 126 kb)

Additional file 8: Excel Spreadsheet including all data in this manuscript. (XLSX 314 kb)

## Abbreviations

2,4-D: 2,4-dichlorophenoxyacetic acid
BAP: 6-benzylaminopurine
FDA: Fluorescein diacetate
FIL2: Inbred population of *Panicum hallii* var. *filipes*
HAL2: Inbred population of *Panicum hallii* var. *hallii*
MS: Murashige and Skoog Medium [30]
MSB: MS supplemented with 13.3 μM BAP, 3% maltose, and 2 gL^−1^ Phytagel
PAHAF: *Panicum hallii* var. *filipes*
PAHAH: *Panicum hallii* var. *hallii*
PI: Propidium iodide
