# Supplementary material for: Improved tissue culture conditions for the emerging C4 model Panicum hallii
Source: BMC Biotechnol. 2017 Apr 27;17:39. doi: 10.1186/s12896-017-0359-0 (PMC5408410; doi:10.1186/s12896-017-0359-0)
Supplement: Supplementary file 1 — Definition of all media used for callus induction. (PDF 121 kb) [file 12896_2017_359_MOESM1_ESM.pdf]

Supplementary Table 1. Definition of all media used for callus induction.

		AA	KM8	LP9	MS-OG	MS-BH	MS-PM	MP-PAH	MP	MS-SEO	MS-SC	MS-Sucrose	MS-Maltose	NB
Macro (mg L^{-1})	(NH4)2SO4	130		460										460
	CaCl2*2H2O	150	600	370	590	590	590	590	590	590	590	590	590	380
	KCl		300											
	KH2PO4		160	390	1700	1700	1700	1700	1700	1700	1700	1700	1700	390
	KNO3	2500	1900	2800	1900	1900	1900	1900	1900	1900	1900	1900	1900	2800
	MgSO4*7H2O	1500	300	1300	370	370	370	370	370	370	370	370	370	1300
	NaH2PO4*H2O	150												
	NH4NO3		600		1600	1600	1600	1600	1600	1600	1600	1600	1600	
Iron (mg L^{-1})	FeSO4*7H2O	28	28	29	29	29	29	29	29	29	29	29	29	18
	Na2 EDTA	37	47	41	41	41	41	41	41	41	41	41	41	26
Micro (mg L^{-1})	CuSO4*5H2O	0.025	0.025	0.024	0.025	0.025	0.025	0.025	0.025	0.025	0.025	0.025	0.025	0.025
	H3BO3	3	3	3	6.2	6.2	6.2	6.2	6.2	6.2	6.2	6.2	6.2	3
	KI	0.75	0.76	0.76	0.83	0.83	0.83	0.83	0.83	0.83	0.83	0.83	0.83	0.75
	MnSO4*H2O	8.9	10	6.8	15	15	15	15	15	15	15	15	15	6.8
	Na2MoO4*2H2O	0.24	0.24	0.24	0.24	0.24	24	0.24	0.24	0.24	0.24	0.24	0.24	0.24
	ZnSO4*7H2O	2	3.2	2	8.6	8.6	8.6	8.6	8.6	8.6	8.6	8.6	8.6	2

Supplementary Table 1. Continued.

		AA	KM8	LP9	MS-OG	MS-BH	MS-PM	MP-PAH	MP	MS-SEO	MS-SC	MS-Sucrose	MS-Maltose	NB
Organics (mg L^{-1})	BD Bacto Casamino Acids (g/L)			0.5										0.3
	Aspartic Acid	0.27												
	Calcium Pantothenate		1.0											
	Cholecalciferol		0.01											
	Choline Chloride		1.0											
	Citric Acid		40											
	Cyanocobalamin		0.01											
	D-Biotin		0.01											
	DL-Malic Acid		40											
	Folic Acid		0.4											
	Fumaric Acid		40											
	Glycine	7.5												
	Inositol	100	100	100	100	100	100	100	100	100	100	100	100	100
	L-Ascorbic Acid		2											
	L-Cysteine				40						34			
	L-Arginine	170												
	L-Glutamine	880		500							500			500

Supplementary Table 1. Continued.

		AA	KM8	LP9	MS-OG	MS-BH	MS-PM	MP-PAH	MP	MS-SEO	MS-SC	MS-Sucrose	MS-Maltose	NB
Organics (mg L^{-1})	L-Proline	2000		500	300			2000	2000			2000	2000	
	L-Tryptophan						42				82			
	Nicotinic Acid	1.0	1.0	1.0	1.0	1.0	1.0	1.0	1.0	1.0	1.0	1.0	1.0	1.0
	p-Aminobenzoic Acid		0.02											
	Pyruvic Acid, Potassium Salt		20											
	Pyridoxine HCl	1.0	1.0	1.0	1.0	1.0	1.0	1.0	1.0	1.0	1.0	1.0	1.0	1.0
	Niacinamide		1.0											
	Riboflavin		0.2											
	Sodium pyruvate		20											
	Thiamine HCl	10	10	10	10	10	10	10	10	10	10	10	10	10
Sugars (g L^{-1})	Maltose							30	30		30	30		
	Sucrose	20	30	30	30	30	30			30			30	30

Supplementary Table 1. Continued.

		AA	KM8	LP9	MS-OG	MS-BH	MS-PM	MP-PAH	MP	MS-SEO	MS-SC	MS-Sucrose	MS-Maltose	NB
Hormones (µM)	2,4-Dichlorophenoxyacetic acid (2,4-D)	0.99	4	5	3	2	2.5	2.2	5	3.6	6.7	5	5	2
	6-Benzylaminopurine (BAP)				0.045			2.3	0.5		1.4	0.5	0.5	
	GA3	0.1												
	IAA						1.0							
	Kinetin	0.2					0.5							
	pH		5.7	5.8	5.8	5.8	5.8	5.8	5.8	5.8	5.7	5.8	5.8	5.8
