# Supplementary material for: Improved tissue culture conditions for the emerging C4 model Panicum hallii
Source: BMC Biotechnol. 2017 Apr 27;17:39. doi: 10.1186/s12896-017-0359-0 (PMC5408410; doi:10.1186/s12896-017-0359-0)
Supplement: Supplementary file 2 — A comparison of regeneration media used. (PDF 10 kb) [file 12896_2017_359_MOESM2_ESM.pdf]

Supplementary Table 2. A comparison of regeneration media used.

Component	REG	REG-SEO	REG-R	REG-SEO-R	Diet MSO
Basal Salts	MS	MS	MS	MS	MS
Vitamins	B5	MS	B5	MS	MS
Maltose (g/L)	30	30			
Naphathalene Acetic Acid (NAA) (µM)		26	26	26	
N-phenyl-N'-[(1,2,3,-thidiazol-5-yl) urea (TDZ)		4.5			
Gibberellic Acid (GA3) (µM)	1.4		1.4		
6-Benzylaminopurine (BAP) (µM)	177.6				
pH	5.8	5.8	5.8	5.8	5.8
