# Supplementary material for: Improved tissue culture conditions for the emerging C4 model Panicum hallii
Source: BMC Biotechnol. 2017 Apr 27;17:39. doi: 10.1186/s12896-017-0359-0 (PMC5408410; doi:10.1186/s12896-017-0359-0)
Supplement: Supplementary file 3 — Effect of 2,4-D on callus growth and type. (a) Total weight in grams of callus after 35 days of 2,4-D treatment. (b) Type of callus induced on each treatment. Data represent three replicates of 33 callus pieces per replicate. ANOVA test showed differences (p < 0.05). Mean separation was analyzed using Tukey’s HSD. Error bars represent the standard error of the mean. (PDF 300 kb) [file 12896_2017_359_MOESM3_ESM.pdf]

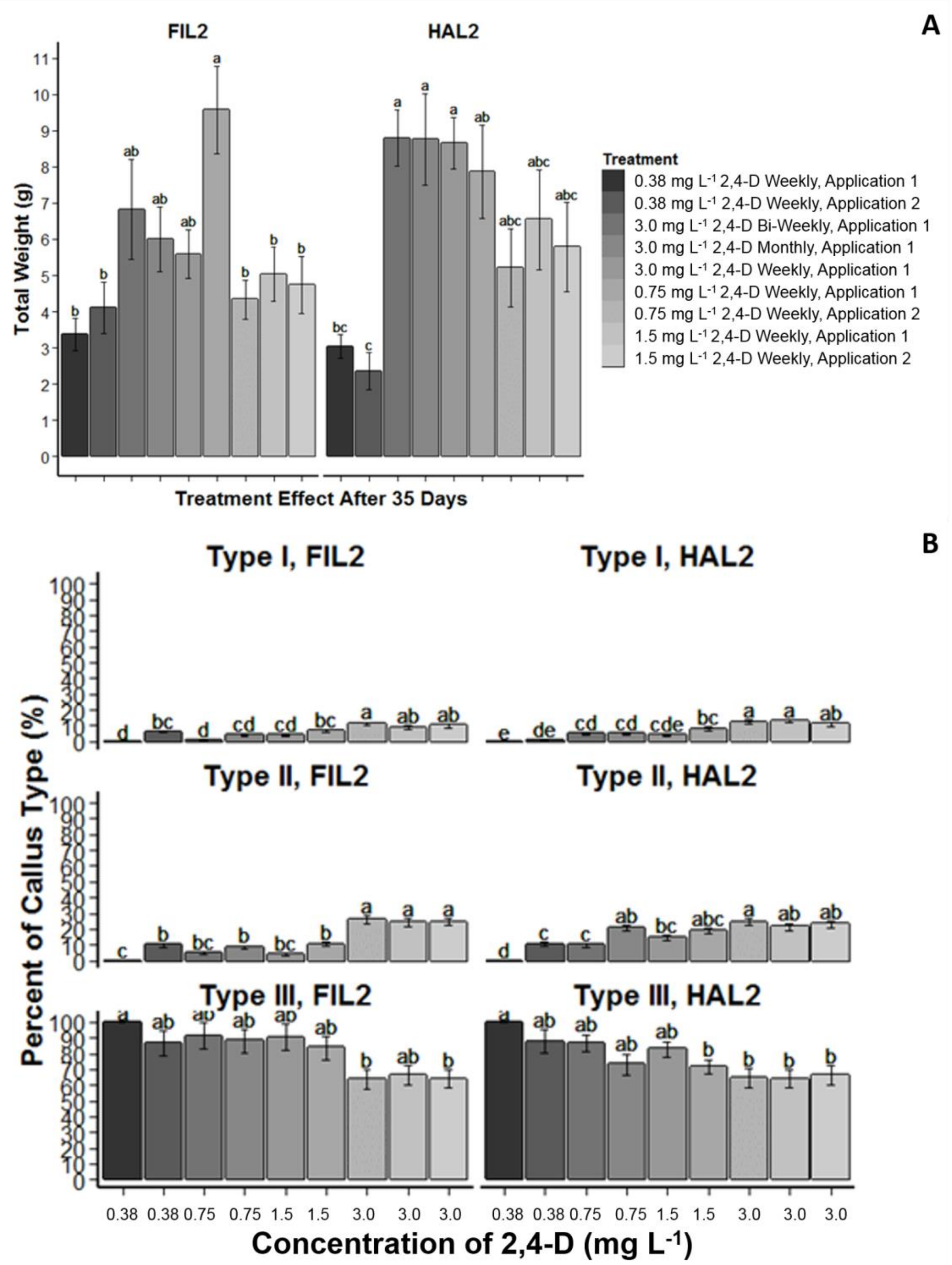

Supplementary Figure S1. A comparison of auxin concentration on total mean area of callus induced after 35 days. Populations and days were analyzed separately under a one-way ANOVA controlling for auxin. ANOVA test showed differences among treatments for both HAL2 and FIL2 populations ($p<0.01$). Mean separation was analyzed **with Tukey's Honest Significant Difference (HSD). These data represent three r**eplicates of 33 callus pieces per replicate.
