# Supplementary figures and images for: Improved tissue culture conditions for the emerging C4 model Panicum hallii
Source: BMC Biotechnol. 2017 Apr 27;17:39. doi: 10.1186/s12896-017-0359-0 (PMC5408410; doi:10.1186/s12896-017-0359-0)

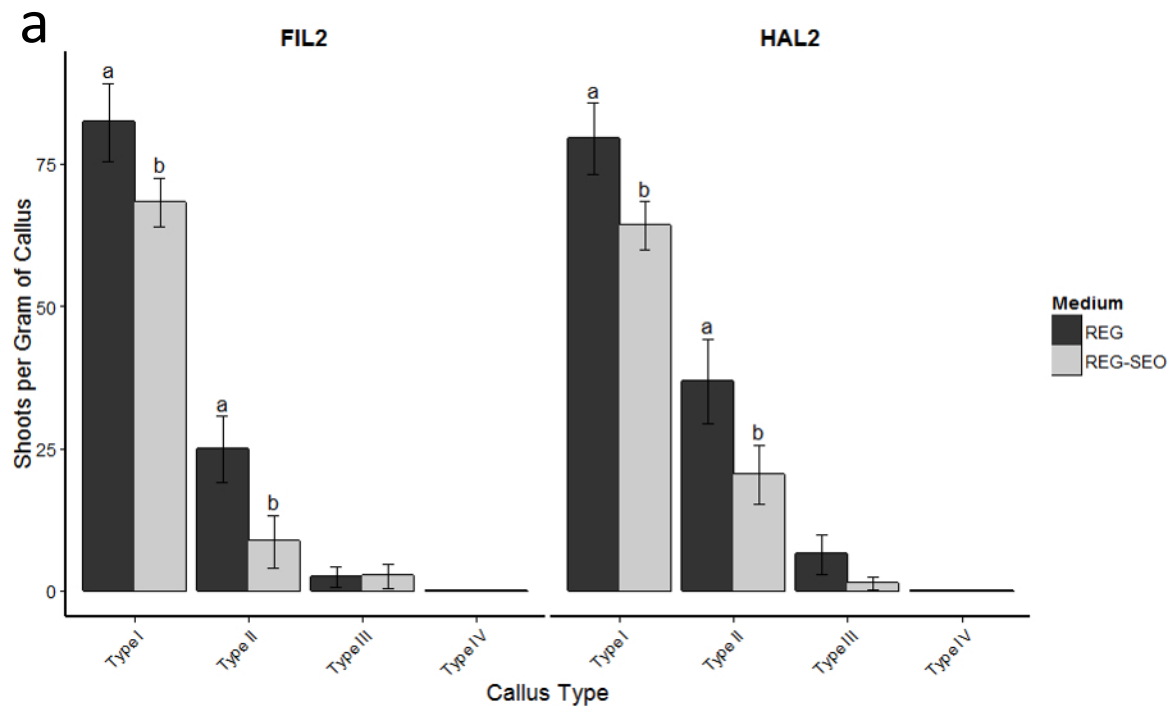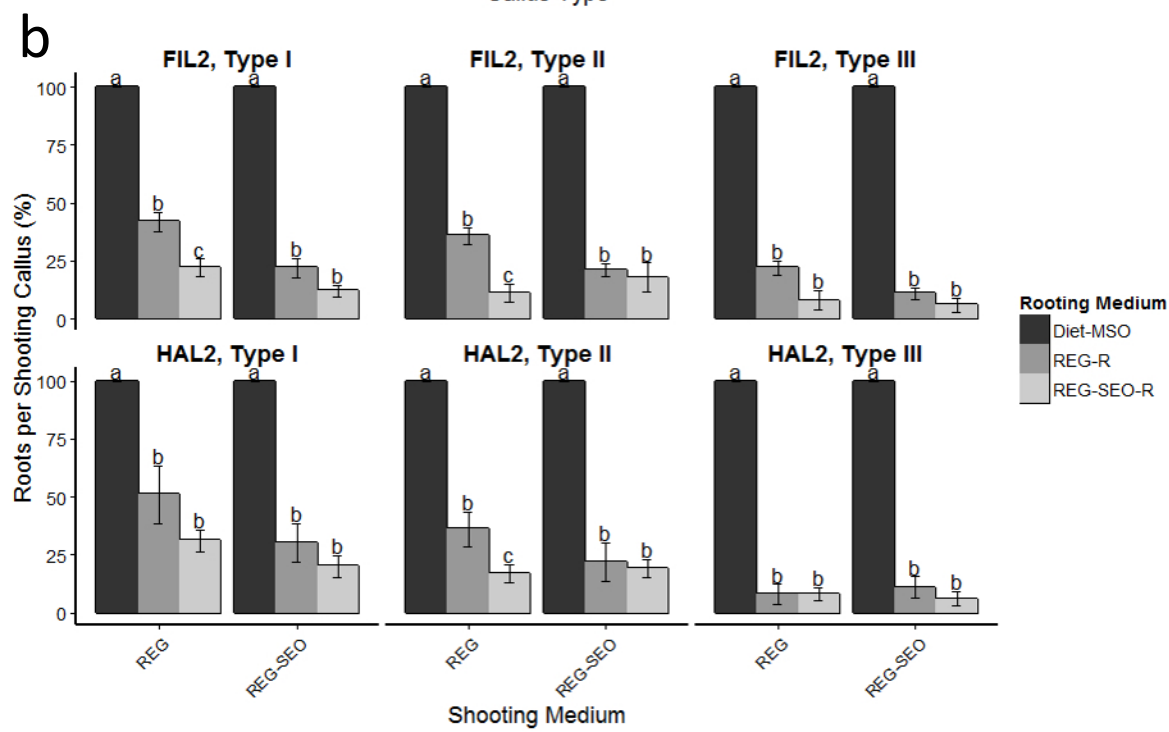

Supplement: Supplementary file 4 — Comparison of callus regeneration by callus type and regeneration media. (a) Regeneration efficiency by callus type. (b) Rooting efficiency based on callus types and regeneration media. Populations and callus types were analyzed separately under a one-way ANOVA controlling for shoot regeneration medium. Mean separation was analyzed using Tukey’s HSD. Error bars represent the standard error of the mean. (PDF 340 kb) [file 12896_2017_359_MOESM4_ESM.pdf]

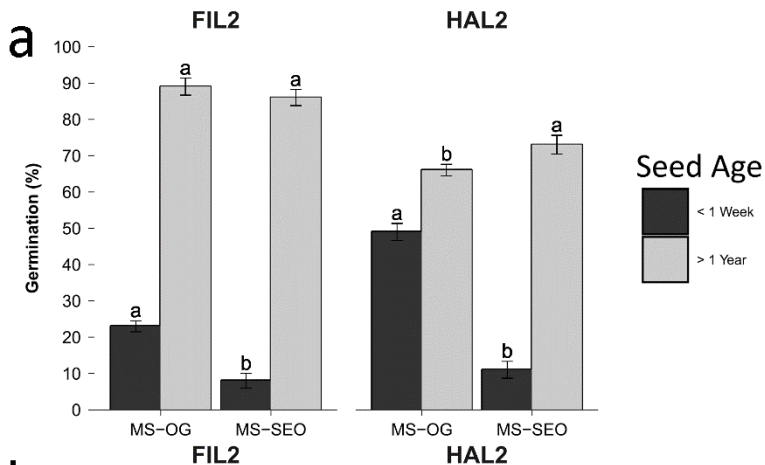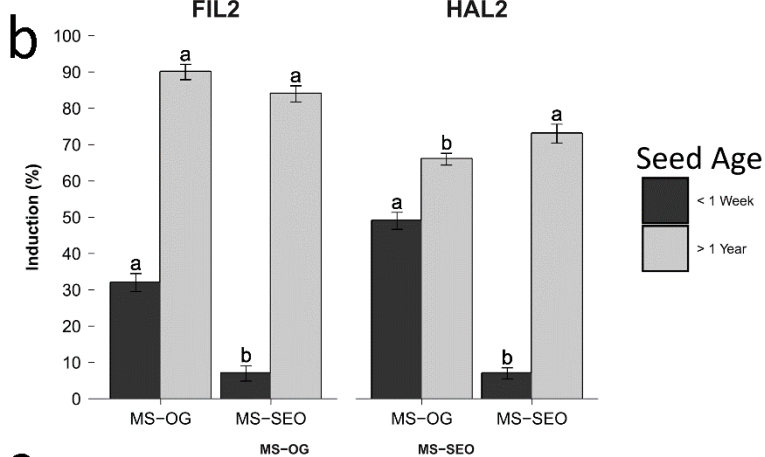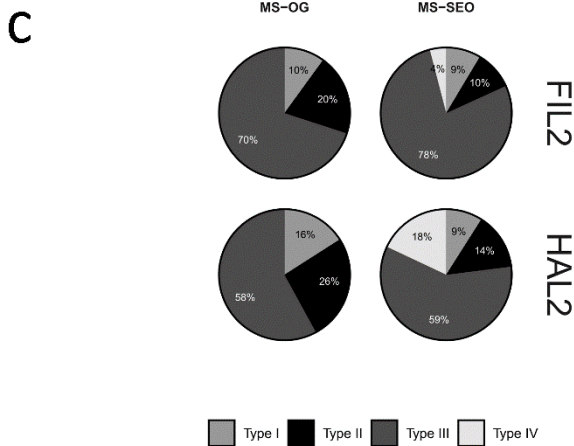

Supplement: Supplementary file 5 — A comparison of FIL2 and HAL2 callus weight change after prolonged culture on MS-OG media. Each week was analyzed separately under a one-way ANOVA controlling for population. ANOVA test showing differences among populations are marked with an asterisk (p < 0.01). These data represent ten replicates of three grams of callus at each subculture. (PDF 157 kb) [file 12896_2017_359_MOESM5_ESM.pdf]

# FIL2

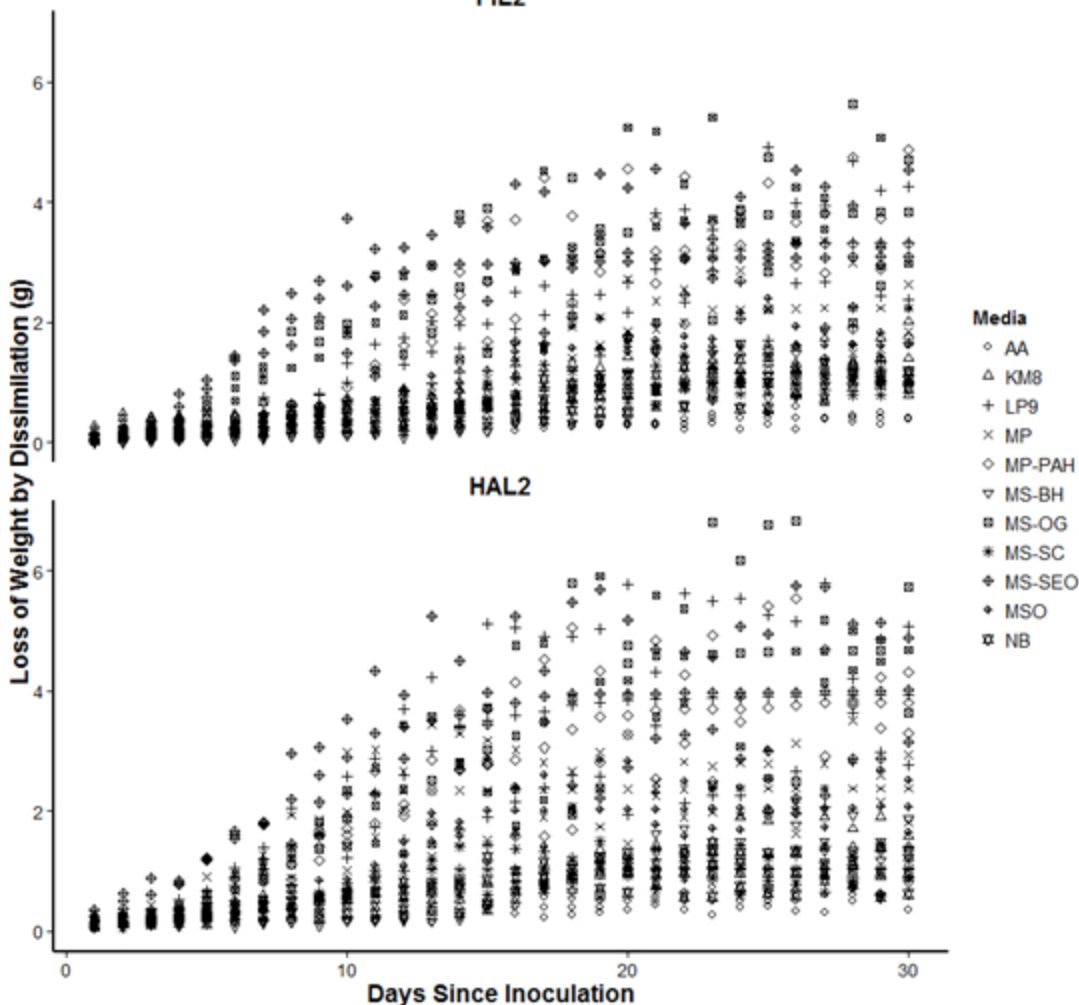

Supplement: Supplementary file 6 — Dissimilation curve of suspension cell cultures of P. hallii. Each point represents one replicate of each measurement. (PDF 131 kb) [file 12896_2017_359_MOESM6_ESM.pdf]

**FIL2**

**HAL2**

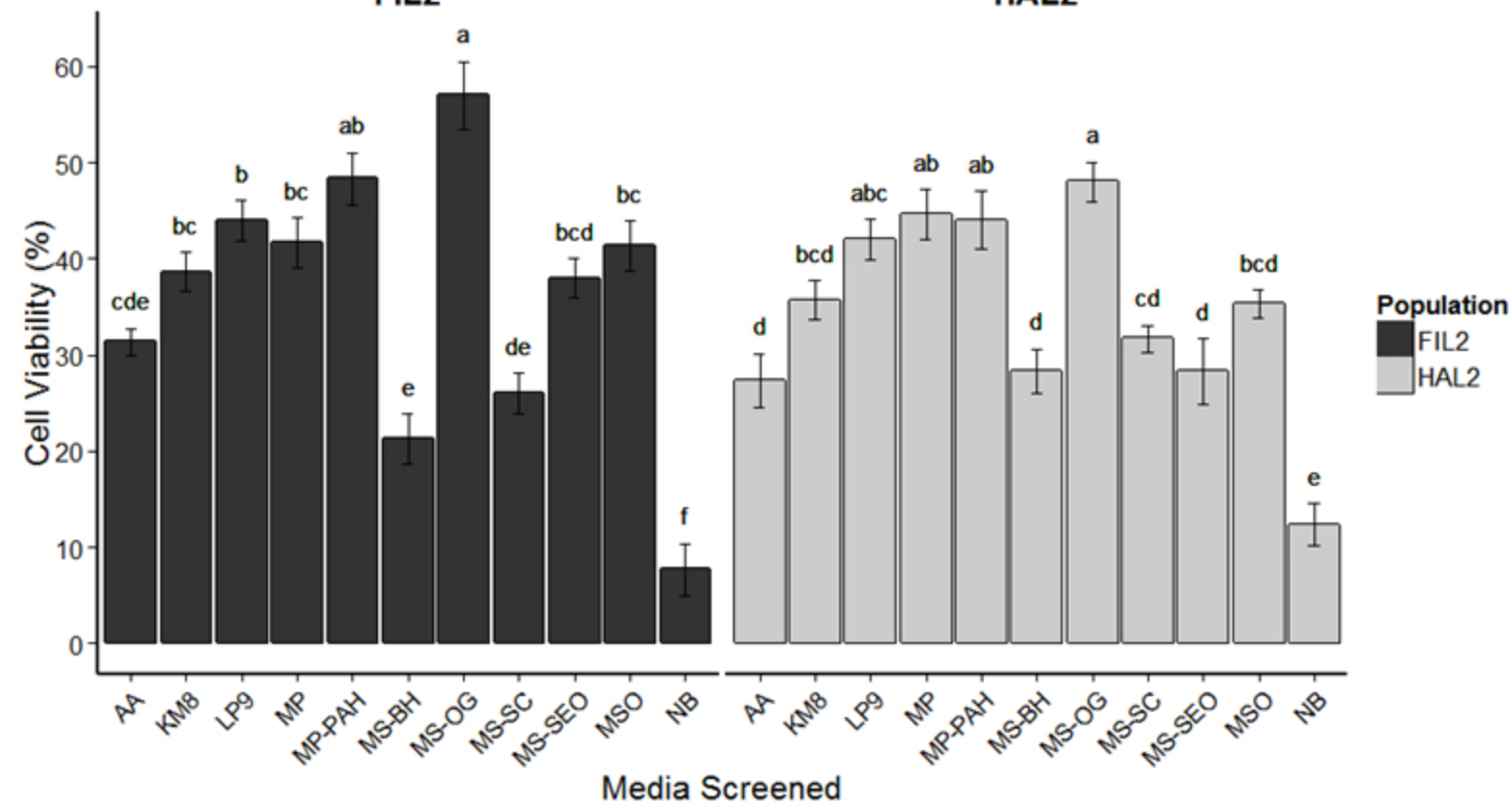

Supplement: Supplementary file 7 — Cell viability of cell suspension cultures as measured by dual staining with FDA and PI. Populations were analyzed separately under a one-way ANOVA controlling for medium (p < 0.05). Mean separation was analyzed using Tukey’s HSD. Data represent two technical replicates of three flasks. Error bars represent the standard error of the mean. (PDF 126 kb) [file 12896_2017_359_MOESM7_ESM.pdf]
